# Deep learning predicts hip fracture using confounding patient and healthcare variables

**DOI:** 10.1038/s41746-019-0105-1

**Published:** 2019-04-30

**Authors:** Marcus A. Badgeley, John R. Zech, Luke Oakden-Rayner, Benjamin S. Glicksberg, Manway Liu, William Gale, Michael V. McConnell, Bethany Percha, Thomas M. Snyder, Joel T. Dudley

**Affiliations:** 1 Verily Life Sciences LLC, South San Francisco, CA USA; 2 Institute for Next Generation Healthcare, Icahn School of Medicine at Mount Sinai, New York, NY USA; 3 Department of Genetics and Genomic Sciences, Icahn School of Medicine at Mount Sinai, New York, NY USA; 4 Department of Medicine, California Pacific Medical Center, San Francisco, CA USA; 5 School of Public Health, The University of Adelaide, Adelaide, South Australia Australia; 6 Bakar Computational Health Sciences Institute, University of California, San Francisco, CA USA; 7 School of Computer Sciences, The University of Adelaide, Adelaide, South Australia Australia; 8 Division of Cardiovascular Medicine, Stanford School of Medicine, Stanford, CA USA

**Keywords:** Radiography, Computer science, Statistics

## Abstract

Hip fractures are a leading cause of death and disability among older adults. Hip fractures are also the most commonly missed diagnosis on pelvic radiographs, and delayed diagnosis leads to higher cost and worse outcomes. Computer-aided diagnosis (CAD) algorithms have shown promise for helping radiologists detect fractures, but the image features underpinning their predictions are notoriously difficult to understand. In this study, we trained deep-learning models on 17,587 radiographs to classify fracture, 5 patient traits, and 14 hospital process variables. All 20 variables could be individually predicted from a radiograph, with the best performances on scanner model (AUC = 1.00), scanner brand (AUC = 0.98), and whether the order was marked “priority” (AUC = 0.79). Fracture was predicted moderately well from the image (AUC = 0.78) and better when combining image features with patient data (AUC = 0.86, DeLong paired AUC comparison, *p* = 2e-9) or patient data plus hospital process features (AUC = 0.91, *p* = 1e-21). Fracture prediction on a test set that balanced fracture risk across patient variables was significantly lower than a random test set (AUC = 0.67, DeLong unpaired AUC comparison, *p* = 0.003); and on a test set with fracture risk balanced across patient and hospital process variables, the model performed randomly (AUC = 0.52, 95% CI 0.46–0.58), indicating that these variables were the main source of the model’s fracture predictions. A single model that directly combines image features, patient, and hospital process data outperforms a Naive Bayes ensemble of an image-only model prediction, patient, and hospital process data. If CAD algorithms are inexplicably leveraging patient and process variables in their predictions, it is unclear how radiologists should interpret their predictions in the context of other known patient data. Further research is needed to illuminate deep-learning decision processes so that computers and clinicians can effectively cooperate.

## Introduction

An estimated, 1.3 million hip fractures occur annually and are associated with 740,000 deaths, and 1.75 million disability-adjusted life years.^1^ The chance of death in the 3 months following a hip fracture increases by fivefold for women and eightfold for men, relative to age- and sex-matched controls.^2^ When a middle-aged or elderly patient presents with acute hip pain and fracture is suspected, clinical guidelines recommend first ordering a hip radiograph.^3^ However, not all fractures are detectable on radiographs.^4,5^ If a patient with high clinical suspicion of fracture has a negative or indeterminant radiograph, then it is usually appropriate to follow-up with a pelvic MRI.^3^ Fractures are the most commonly missed diagnosis on radiographs of the spine and extremities, and the majority of these errors are perceptual (i.e., a radiologist not noticing some abnormality as opposed to misinterpreting a recognized anomaly).^6^

Statistical learning models can both detect fractures and help radiologists detect fractures. Past studies used machine learning (ML) to identify combinations of hand-engineered features associated with fracture, and more recent studies used deep learning (DL) to discover hierarchical pixel patterns from many images with a known diagnosis. Most studies detect fracture in algorithm-only systems.^7–9^ Kasai et al. performed a clinical trial to study how algorithms can augment radiologists and found radiologists were significantly better at detecting vertebral fractures when aided by an ML model that had a standalone sensitivity of 81%.^10^ Convolutional Neural Networks (CNNs), the DL models best suited for image recognition, have recently been used to detect fracture in the appendicular skeleton, including wrists,^11^ shoulders,^12^ and hands and feet.^13^ Gale et al. developed the only previously reported hip fracture detector using DL; their model achieved an area under the receiver-operating curve (AUC) of 0.994.^14^ These academic DL reports compared isolated image model performance against humans, but none tested whether algorithms could aid human diagnosis. In contrast, the company Imagen Technologies’ OsteoDetect DL system reported improving humans from an unaided AUC 0.84 to AUC 0.89 (improvement of 0.05, 95% CI 0.02–0.08), according to a letter from the FDA (https://www.accessdata.fda.gov/cdrh_docs/pdf18/DEN180005.pdf). Deep-learning studies on various image-based fracture prediction tasks have been published, but they do not consider patient and hospital covariates, or how algorithms can augment human decision processes.

Statistical learning algorithms sometimes learn unintended or unhelpful patterns contained in the model training data. Diverse examples for common DL applications include gender being differentially classified on photographs of the face depending on a person’s race^15^ as well as gender detection on photographs of the outer eye leveraging disproportionate use of mascara.^16^ Language-processing algorithms learned to perpetuate human prejudices from the text to the web.^17^ An observational ML study on healthcare labs found that the timing of a lab order (day of week, hour of day, and time since prior order) was more predictive of patient mortality than biological signal (the measured value and abnormal flag) for 68% of labs associated with mortality.^18^ All observational datasets have some bias and confounding.^19^ A confounding variable (e.g., time since prior lab order, or which scanner in a hospital is used to acquire a radiograph) is associated with both an explanatory variable (e.g., acuity of a patient’s illness, or a patient’s clinically predicted risk of fracture) and an outcome (e.g., mortality, or the likelihood of a radiograph’s pixels containing patterns suggestive of fracture). When researchers overlook a confounding variable, the purported explanation for an outcome is distorted. Patient and healthcare process variables impress patterns into observational healthcare data, and these patterns can be learned by statistical learning algorithms.

DL has previously been shown to detect patient demographics and clinical variables from fundoscopy images.^20^ We previously showed that DL can learn individual hospital sources in a multisite trial and leverage this for disease detection, which leads to inconsistent performance when deployed to new hospitals.^21^ Here, we perform a comprehensive analysis of what patient and hospital process variables DL can detect in radiographs and whether they contribute to the inner workings of a fracture detection model. We group variables into four classes: disease (fracture), image (radiograph pixels), patient (sex, age, body mass index, pain, and recent fall), and hospital processes (department, scanner model, scanner manufacturer, laterality, study date [and day of week], order priority, technician, radiologist, radiation dose, time from image order to acquisition, time from image acquisition to initial interpretation, and time from image acquisition to final interpretation). See Supplementary Table 1 for descriptions of the scalar (non-image) variables. We use these scalar variables to develop multivariate models and to create matched patient cohorts to test image-only models. We refer to multivariate models that combine image features with scalar variables as “multimodal” models. To assess the suitability for image-only models augmenting human interpretations, we experiment with Naive Bayes model ensembles. We reanalyze test data from the best published hip fracture model by Gale et al. and conclude by highlighting design of clinical experiments and strategies to mitigate the susceptibility of models to confounding variables.

## Results

### Dataset and unsupervised analysis

**Fig. 1:**
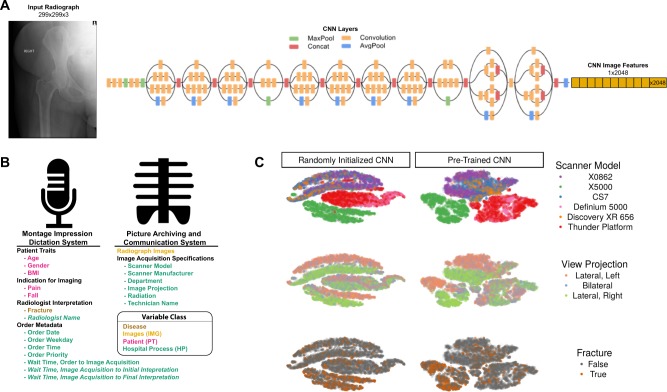
The main source of variation in whole radiographs is explained by the device used to capture the radiograph. **a** Schematic of the inception v-3 deep learning model used to featurize radiographs into an embedded 2048-dimensional representation. Inception model architecture schematic derived from https://cloud.google.com/tpu/docs/inception-v3-advanced. **b** Data were collected from two sources. Variables were categorized as pathology (gold), image (IMG, yellow), patient, (PT, pink), or hospital process (HP, green). Italicized variables are not known at the time of image acquisition and are not used as explanatory variables. **c** The distribution of radiographs projected into clusters by t-Distributed Stochastic Neighbor Embedding (t-SNE) and designates how the unsupervised distribution of clusters relates to hip fracture and categorical variables

We collected 23,602 hip radiographs from 9024 patients and associated patient and hospital process data from the medical imaging and clinician dictation databases, of which 23,557 were used to train and test (3:1 split) Convolutional Neural Networks (CNNs) (Supplementary Fig. 1). The prevalence of fracture was 3% (779/23,602), and patients with fractures were more likely to report a recent fall and less likely to report pain (Supplementary Table 3). Extracted variables are separated into disease (i.e., fracture), image (IMG), patient (PT), or hospital process (HP) variables, and only those known at the time of image acquisition are used as explanatory variables (Fig. 1b). We used the inception-v3 model architecture (Fig. 1a) and computed image features for all radiographs with randomly initialized model weights and weights pre-trained on everyday images. The pre-trained model takes a 299 × 299 pixel input and computes 2048 8 × 8 feature maps, and we averaged each feature map to get a 2048-dimensional feature vector. Clustering analyses showed that the greatest source of variation between radiographs is the scanner that captured the image, and within each scanner the laterality forms further discrete clusters (Fig. 1c). The image feature matrix demonstrates a concomitant clustering by these image acquisition features (Supplementary Fig. 2). The scanner model is the best predictor of the first Principal Component (PC) (R^2^ = 0.59) and eight of the first ten PCs (Supplementary Table 4).

### Modeling fracture, patient traits, and hospital process variables

**Fig. 2:**
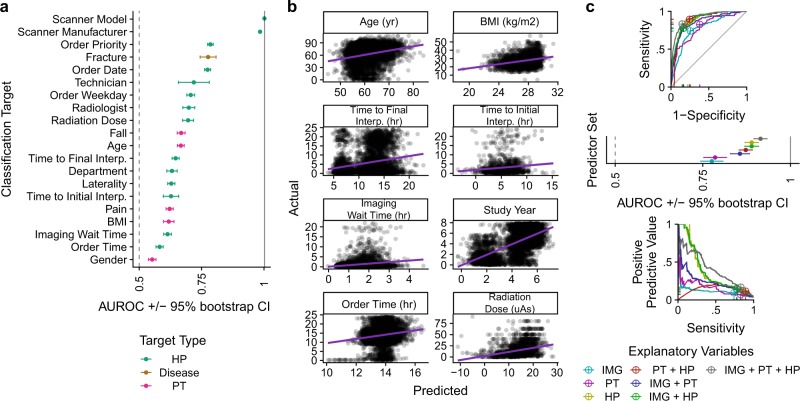
Deep-learning predicts all patient and hospital processes from a radiograph. **a** Deep-learning image models to predict binarized forms of 14 HP variables, 5 PT variables, and hip fracture. Error bars indicate the 95% confidence intervals of 2000 bootstrapped samples. **b** Deep-learning regression models to predict eight continuous variables from hip radiographs. Each dot represents one radiograph, and the purple lines are linear models of actual versus predicted values. **c** ROC, ROC^+/−^ bootstrap confidence intervals, and precision recall curves for deep-learning models that predict fracture based on combinatorial predictor sets of IMG, PT, and HP variables. Crosshairs indicate the best operating point on ROC and PRC curves

We transformed all scalar variables into binary factors and trained logistic regression models for fracture, PTs, and HPs as described in detail in the Methods section (see Supplementary Table 2 for original and binarized variable representations). All 20 of 20 image models were significantly better than random (DeLong 95% AUC CI > 0.5) (Fig. 2a, Supplementary Table 5). Hip fracture was detected with AUC 0.78 (95% CI: 0.74–0.81), and the best detected secondary targets were the device that took the scan (AUC 1, CI 1–1), scanner manufacturer (AUC 0.98, 95% CI 0.98–0.99), and whether the image was ordered as a high priority (AUC 0.79, 95% CI 0.77–0.80). The difference in performance across targets is not explained by differences in the total sample size or the number of examples in the smaller class (Supplementary Fig. 3). Most of the patient and hospital process factors (15/19) were themselves significantly associated with fracture (Fisher’s exact test, *p* < 0.05); and, after stratifying by device, the other four covariates were associated with fracture on at least one device (Supplementary Fig. 4). The best-predicted continuous variable is the year the scan was ordered (R^2^ = .39) (Fig. 2b; Supplementary Table 6).

### Multimodal deep-learning models

We then compared combinatorial sets of IMG, PT, and HP features for fracture prediction. Missing PT + HP data were imputed as described in Supplementary Methods. Multivariate model performance metrics are provided in Supplementary Tables 7–8, and operating point independent statistics are shown in Fig. 2c. Fracture was better predicted by HP features (AUC 0.89, 95% CI 0.87–0.91) than either IMG features (AUC 0.78, 95% CI 0.74–0.81) or PT features (AUC 0.79, 95% CI 0.75–0.82). Adding IMG to the HP set did not improve performance (DeLong paired AUC comparison, *p* = 0.97). The best predictor set was the full set of IMG + PT + HP (AUC 0.91, 95% CI 0.90–0.93).

### Evaluating CNNs on matched patient populations

**Fig. 3:**
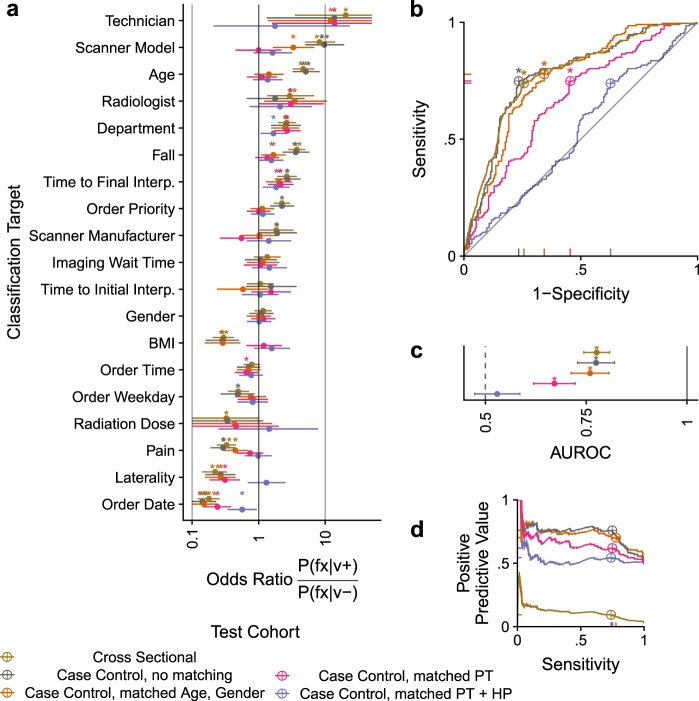
Deep-learning hip fracture from radiographs is successful until controlling for all patient and hospital process variables. **a** The association between each metadata variable and fracture, colored by how the test cohort is sampled. (*) indicate a Fisher’s Exact test with *p* < 0.05. (**b**) ROC and (**d**) precision recall curves for the image-classifier tested on differentially sampled test sets. The best operating point is indicated with crosshairs. (*) represents a 95% confidence interval that does not include 0.5. **c** Summary of (**b**) with 95% bootstrap confidence intervals

We sought to disentangle the ability of a CNN to directly detect fracture versus indirectly predicting fracture by detecting confounding variables associated with fracture. We manipulate the associations between confounders and fracture by subsampling the full (cross-sectional) test set in a case–control fashion (see the Methods section for case–control subsampling details). By selecting non-fracture cases (controls) with similar distributions of patient and hospital process variables as fracture cases, the conditional probability of fracture becomes constant across different patient and hospital process profiles. We derived test sets with fracture risk balanced across demographics (age, gender), PT variables, or PT + HP variables, and found that with increasingly comprehensive matching, the number of fracture-associated covariates consistently decreased (Table 1, Fig. 3a).

**Table 1:** Cohort Characteristics after various Sampling Routines

Cohort	cs-train	cs-test	cc-Random	cc-Dem	cc-Pt	cc-PtHp
Sampling	Cross-sectional	Cross-sectional	Case–control	Case–control	Case–control	Case–control
Matching	NA	NA	NA	Age + gender	PT	PT + HP
Partition	Train	Test	Test	Test	Test	Test
No. of radiographs	17,587	5970	416	405	416	411
No. of patients	6768	2256	275	252	217	186
No. of scanners	11	11	10	9	8	6
No. of scanner manufacturers	4	4	4	4	4	4
Age, mean (SD), years	61 (22)	61 (22)	67 (24)	75 (20)	75 (21)	74 (19)
Female frequency, no. (%)	11,647 (66)	3873 (65)	260 (62)	249 (61)	263 (63)	253 (62)
Fracture frequency, no. (%)	572 (3)	207 (3)	207 (50)	207 (51)	207 (50)	207 (50)
BMI, mean (SD)	28 (7)	28 (7)	25 (5)	25 (5)	24 (5)	24 (4)
Fall frequency, no. (%)	3214 (18)	1139 (19)	133 (32)	160 (40)	174 (42)	165 (40)
Pain frequency, no. (%)	9010 (51)	2960 (50)	164 (39)	137 (34)	117 (28)	104 (25)

**Fig. 4:**
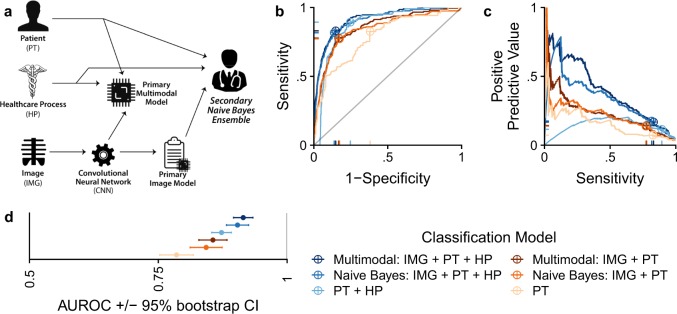
Deep learning a compendium of patient data by directly combining image features, PT, and HP variables in multimodal models, or by secondarily ensembling image-only model predictions with PT and HP variables. **a** experiment schematic demonstrating the CAD simulation scenario wherein a physician secondarily integrates image-only and other clinical data (as modeled in a Naive Bayes ensemble). **b** ROC and (**c**) precision recall curves for classifiers tested on differentially sampled test sets. The best operating point is indicated with crosshairs. **d** Summary of (**b**) with 95% bootstrap confidence intervals

We then evaluated the image-only classifier for fracture on each test set (Fig. 3b–d; Supplementary Tables 9–10). The area under the Precision Recall Curve (PRC) is dependent on the disease prevalence, and since the original population had a 3% fracture prevalence, but case–control cohorts have a 50% prevalence, the PRC is significantly higher for case–control cohorts. Random subsampling had no effect on the primary evaluation metric, AUC (0.78 vs. 0.77, DeLong unpaired AUC comparison, *p* = 0.96). Model performance was consistent after matching by demographics (AUC = 0.76, *p* = 0.65), but significantly lower after matching by all patient variables (AUC = 0.67, *p* = 0.003). When evaluated on a test cohort matched by all covariates, the fracture detector was no longer better than random (AUC = 0.52, 95% CI 0.46–0.58) and significantly worse than when assessed on all other test cohorts.

### Evaluating effect of confounding variables on model of Gale et al.

We repeated this case–control testing experiment on the model previously reported by Gale et al.^14^ There were fewer covariates to match patients by (Supplementary Tables 11–12, Supplementary Fig. 5A), and the fracture detection results were robust across case–control subsampling routines (Supplementary Fig. 5B–D, Supplementary Tables 13–14). Performance on a randomly subsampled test (AUC 0.99, 95% CI 0.99–1.0) was similar (DeLong unpaired AUC comparison, *p* = 0.14) to the fully matched cohort (AUC 0.99, 95% CI 0.98–1.0).

### Secondary evidence integration from image models and clinical data

Computer-Aided Diagnosis (CAD) tools provide clinicians with Supplementary Information to make diagnostic decisions. We have shown that deep-learning models benefit from leveraging statistical relationships between fracture and patient and hospital process variables when the algorithms are tested in isolation, but we have not studied the impact this has under a CAD use scenario. We simulate a CAD scenario by training separate models that predict fracture with individual sets of predictors and then train ensembles or multimodal models that use a combination of predictor sets (Fig. 4a). To simulate a clinician’s reasoning, we use a Naive Bayes ensemble that integrates the CNN’s image prediction with the likelihood of disease based on covariates. We opine that this ensemble model is reflective of how a clinician might use an image model—without any knowledge of the inner workings of predictive models, a clinician would be unable to control for the fact that available models may be basing their predictions on overlapping information. For a positive control, we train a multimodal model which encodes the interdependencies between image and covariate predictor sets.

Multimodal models trained directly on IMG + PT and IMG + PT + HP outperform models without image data (Fig. 4b–d). Secondary integration of IMG predictions with PT predictions (Naive Bayes AUC 0.84, 95% CI 0.81–0.87) is better (DeLong paired AUC comparison, *p* = 2e-8) than considering only PT (AUC 0.79, 95% CI 0.75–0.82), but worse (*p* = 0.01) than directly combining IMG + PT data (multimodal AUC 0.86, 95% CI 0.83–0.89). Similarly, secondary integration of IMG predictions with PT + HP predictions (Naive Bayes AUC 0.90, 95% CI 0.88–0.93) is better (DeLong paired AUC comparison, *p* = 5e-11) than considering only PT + HP (AUC 0.87, 95% CI 0.85–0.89), but worse (*p* = 0.004) than directly combining IMG + PT + HP (AUC 0.91, 95% CI 0.90–0.93). Combining an image-only model result with other clinical data improves upon the clinical data alone, but does not reach the performance of directly modeling all data (Supplementary Tables 15–16).

## Discussion

CNNs can use radiograph pixels to predict not only disease but also numerous patient and hospital process variables. Several prior studies demonstrated the ability of CNNs to learn patient traits^20^ and image acquisition specifications,^13,14,22^ but this study systematically compared how deep learning can detect disease, demographics, and image acquisition targets, and use all of these factors to improve prediction performance. We further show that CNNs learn to encode the statistical relationships between patient and hospital process covariates and hip fracture. Despite CNNs directly encoding some of these variables, the direct addition of patient and hospital process variables to image features in multimodal models further boosts predictive performance, while secondarily combining an image model with other variables is less beneficial. This study expands on previous work that incorporates interdependencies between disease comorbidities to improve CNN predictions^23^ by considering the compendium of patient and hospital process variables involved in routine clinical care.

The standard of care a patient receives is based on his or her differential diagnosis and the pre-test probability of disease, and these differences in diagnostic workups can induce structure into healthcare data that is learned by statistical learning algorithms. We reproduced known associations between patient traits and hip fracture (e.g., older age, low body weight).^24^ Because of these known patient associations, middle aged or elderly patients clinically suspected to have a hip fracture should have a follow-up MRI if the X-ray is negative or indeterminant.^3^ The different standards of care based on patients’ global health was previously noted by Agniel et al. to be more predictive than the biological values that were being measured.^18^ They concluded that “Electronic Health Record (EHR) data are unsuitable for many research questions.”

We leveraged case–control matching to eliminate the associations between fracture and PT + HP variables. The ML model of clinical laboratory tests by Agniel et al. had discrete inputs which made biologic and non-biologic signals easily separable (e.g., the time a lab is ordered was an explanatory variable in a healthcare process model, and the measured lab value was used in a pathophysiology model). In contrast, we used deep learning of radiographs and found that DL can detect both biologic and non-biologic signal from the image pixels, which precluded us from simply separating these variables. As an alternative to manipulating each image (e.g., obscure the image pixels that suggest a device’s identity), we used matched subsampling to alter the statistical associations between fracture and PT + HP variables on a population scale. When deep learning rare conditions, it is common to perform class balancing by downsampling normal examples, but this is generally done randomly without considering PT or HP variables. Clinical trials with a case–control study design sometimes down-sample the normal patients with a matching routine to evenly distribute known confounding variables.^25^ After selecting control radiographs that match the PT + HP distributions of fracture cases, the associations between these factors and fracture were blotted out, and the model was no longer able to detect hip fracture. This loss of predictive performance indicates that the model was predicting fracture indirectly through these associated variables rather than directly measuring the image features of fracture.

While most DL models perform random class balancing, exceptions have used demographics for radiograph matching.^26^ However, we found that a richer set of matching variables was required to uncover the dependency on confounding variables. Our model’s performance was consistent after matching demographics, and the dependence on other variables was only revealed when additionally controlled by patient symptoms and hospital process variables. The addition of patient symptoms to demographics matching had the side effect of equating the odds of fracture on the top two devices, consistent with the hypothesis that patients are triaged differently which induces HP biases into the data. Given that DL is able to detect so many variables from the radiographs, balancing just demographics does not suffice to reveal the classification mechanisms that were exposed after balancing with PT + HP variables.

Although our model was dependent on covariates to predict fracture, the previously reported DL model for hip fracture detection by Gale et al. was not. We attribute this to the fact that Gale et al. used different training data and modeling strategies.

The datasets had different sizes, scalar variables, diversity, and label accuracy. Gale et al. had twice as many training examples and a much higher fracture rate (12% vs. 3%). The patient populations came from different clinical settings (Emergency Department [ED] patients in Australia versus ED, inpatients, and outpatients at Mount Sinai Health System [MSHS] New York City [NYC]). Among the Sinai ED patients, the fracture rate was 4%, a one-third of Adelaide’s ED fracture frequency. This may reflect discrepancies in healthcare policy. Australia has free and universal health care, while the US has no free or universal healthcare but Centers for Medicare & Medicaid Services aggressively enforces legislation mandating EDs to evaluate and care for all patients (Emergency Medical Treatment and Labor Act).^27^ The American healthcare policy can result in patients with chronic or low-acuity illness seeking treatment from the ED (especially if they cannot afford care elsewhere). There were stronger associations between fracture and HP covariates at MSHS, possibly because radiographs were collected from a wider range of clinical settings or because NYC has a richer patient diversity or more tragic health disparities. The metadata collected from MSHS was more extensive so patients were matched on additional symptoms and HP variables. The labels for Gale et al. were semi-manually curated, whereas MSHS was solely inferred from clinical notes (see Limitations below).

Gale et al. also used a different deep-learning strategy that leveraged multiple CNN models. Specifically, Gale et al. developed an extra set of labels for the location of the hip joint and trained a preprocessing CNN to crop the whole radiograph and zoom in on this region of interest. Then they feed the resulting hip joint image into a DenseNet classifier to predict fracture. This sequential CNN localization and classification approach has been referred to as using cascaded CNNs. Gale et al. also used a customized DenseNet architecture that allowed them to maintain high resolution of the region of interest (1024 × 1024 pixels, in contrast to the 299 × 299 of the inception v3 model used for our primary experiments).

Our training dataset had 17,587 radiographs, but only 572 of those had a fracture. DL studies on radiographs have used training datasets with magnitudes of different image counts: ~1000,^8,12,28^ ~10,000,^29–32^ ~50,000,^14^ ~100,000,^23,33^ 250,000,^13^ and 1.5 M.^27^ Some of these datasets had class imbalance, with minor classes found in few images: 15,^8^ 32,^30^ 492,^28^ 515,^12^ 695.^11^ Class imbalance can be partly mitigated by data augmentation strategies.^8,12,28,29,33^ Islam et al. report that training a cardiomegaly classifier with fewer than 50 positive examples leads to poor performance, and that performance plateaus at 200 positive examples.^30^ Outside of medical imaging, DL models are commonly trained with larger datasets, on the order of Brestel et al.’s 1.5 M. The limited number of positive examples in this study may be a contributing factor to why the model resorted to leveraging correlated PT and HP data instead of directly learning fracture.

We previously reported that confounding variables are more likely to be leveraged by deep learning in heterogenous datasets and that confounders may be located at the edge of whole radiographs (within the image, but outside of the patient).^21^ Gale et al.’s hip localization step may mitigate the impact of non-biological variables being considered by DL classification models.^21^ Various localization strategies have been employed before applying DL to radiology data. We and others perform whole-image classification since labels can be extracted in a semi-automated fashion from clinical notes.^11,23,28–31,33^ Some investigators manually indicate the region of interest on every single image before “automated detection”^12^ or use heuristics to crop scans.^13,32^ The use of cascaded CNNs to initially segment images requires an additional training dataset, but it has been embraced by several investigators.^14,34–36^ The description of the FDA-approved OsteoDetect suggests that it was not performing classification but trained with pixel-level data to perform pure segmentation. The combination of zooming down to a region of interest and maintaining high resolution of the CNN’s receptive field may ensure that valuable fracture-specific radiographic findings were not lost in the process of image downsampling.

Deep learning is frequently criticized because the predictions lack clear attribution, and people are uneasy about blindly trusting a machine. If a DL model is acting fully autonomously, as has been proposed for retinopathy screening,^37^ then it can benefit from incorporating patient and process variables, and it is inconsequential whether this behavior is explicitly known. However, if the algorithm is intended to provide a radiologist with an image risk score so the radiologist can consider this in addition to the patient’s documented demographics and symptoms, a patient interview and physician exam, then it is undesirable if the CNN is unknowingly exploiting some portion of these data. This behavior creates uncertainty around how much of the CNN’s prediction is new evidence or redundant information. If the clinician presumes an image-only interpretation model is not leveraging patient or process variables, they may consider the evidence as statistically independent, as the Naive Bayes model assumes. For a Naïve Bayes evidence ensemble, this false assumption produces a worse prediction than one based on full knowledge of interdependencies.

Clinicians are better at interpreting images when they consider a patient’s clinical context.^27,38^ As clinicians are frequently considering other evidence sources not available to the model (e.g., clinical history, past imaging), it is important to enable them to synthesize a model’s predictions with a patient’s larger clinical scenario. Multimodal models allow an algorithm’s prediction to be separately attributed to image or PT + HP variables. Patient risk profiles have been created from deep-learning representations of EHR data that compute the risk difference attributable to age, gender, and prior hospitalizations.^39^ With an analogous system for multimodal models, a physician could integrate further context. For example, if a multimodal model explicitly computes the risk differential for fracture due to a women being postmenopausal, a physician could make informed adjustments for those women on hormone replacement therapy.^40^ We used 20 variables that were recorded in the image metadata and radiologists dictation database, but future studies could develop a richer patient representation by incorporating features from the EHR.

Previous image recognition CAD studies may not have considered PT and HP variables because they are inconsistently reported and/or unstructured in the EHR. Better standards for documenting the risk factors a clinician considers during diagnosis could allow more image recognition studies to incorporate multimodal predictor sets.

Several factors limit this study’s input data quality and model predictive performance. First, we did not have gold standard images: plain film radiology is the first line of the diagnostic work-up, but not the gold standard imaging modality for hip fracture detection.^3^ Second, our labels have limited accuracy: we used natural language processing to automatically infer the presence of fracture in a radiographic study from the clinical note. Since the radiologist had multiple images and non-imaging data, a fracture may not be discernible on every image we labeled as fracture, which has been previously reported.^13^ Third, our covariate data have limited accuracy: we imputed missing patient BMIs and HP variables when they were not documented consistently. Fourth, our preprocessing reduces image resolution: we used a pre-trained network, which required us to downscale the images from full resolution to 299 × 299 pixels. We further reduced the detail in the image representation by using a CNN feature dimensionality reduction to constrain the image feature space. Finally, we used a simple fine-tuning strategy and did not optimize model hyperparameters or model selection with a validation set. These image preprocessing and fine-tuning simplifications were done to expedite model training since our investigative questions involved training and comparing many models rather than training a best model.

Further research is needed to investigate sampling biases and generalization in DL observational medical datasets. Pixel-level image annotation can enable pre-segmentation in cascaded networks. Datasets with complete metadata can perform matched experimental designs, but the largest medical radiology datasets that are publicly available do not contain image acquisition specifications or hospital process variables, and we would need to develop more intricate methods to mitigate the influence of non-biological signal in these resources. Genomic analyses have accounted for unmeasured confounding variables via surrogate variable analysis,^41^ factor analysis,^42^ and mixed models.^43^ DL approaches to mitigate undesired signal include adversarial networks^44^ and domain separation networks.^45^

DL algorithms can predict hip fracture from hip radiographs, as well as many patient and hospital process variables that are associated with fracture. Observational medical data contain many biases, and radiographs contain non-biological signal that is predictive of disease, but may not be ideal for computer-aided diagnosis applications. Directly extending DL image models with known covariates can improve model performance, and performing localization steps before classification may mitigate dependence on these covariates. Given that the largest public datasets lack covariate annotations, further research is needed to understand what specific findings are contributing to a model’s predictions and assess the impacts of DL’s incorporation of non-disease signal in CAD applications.

## Methods

### Study design

We collected a new dataset from Mount Sinai Health System (MSHS), and then reanalyzed the previously published results from the University of Adelaide. The University of Adelaide data and deep-learning model methods were previously described^14^ and differ from those used on MSHS data. Below we elaborate on the MSHS dataset and model development methods. Separate models were developed from each site and used to predict fracture on internal test set images. The patient matching and model evaluation described below were applied to both site’s test set predictions. Train-test partition was stratified by patient so no patient had radiographs in both train and test sets.

### Imaging studies

Overall, 23,602 hip radiographs were retrieved from the Picture Archiving Communication System (PACS) in a DICOM file format, of which 23,557 radiographs from 9024 patients were included in the study (see Supplementary Fig. 1). The retrospective study was performed in accordance with ethical regulations and approved by the Icahn School of Medicine at Mount Sinai institutional review board with waived informed consent. Hip radiographs were collected from 2008 to 2016. Image were acquired for routine medical practice from several clinical sites (inpatient 4183, outpatient 3444, ED 7929, NA 8005) on 12 devices manufactured by Fujifilm, GE, Konica, and Philips. All films had an anteroposterior or frog-leg projection, and the laterality was mixed (left, right, or bilateral). The distribution of sample characteristics by device and department are shown in Supplementary Tables 17–18, and the association between departments and devices is depicted in Supplementary Fig. 6.

### Image preprocessing

Radiographs were standardized to a common size and pixel intensity distribution. Image were downsampled and padded to a final size of 299 × 299 pixels. Pixel intensity mean and standard deviation were normalized per-image.

### Scalar variable extraction

Fracture, PT, and HP variables were parsed from two sources: the DICOM file header and clinical notes (see Fig. 1b). The DICOM file headers recorded the image acquisition specifications in a tabular format. The clinical notes recorded patients’ demographics and radiologists’ interpretation times in a tabular format, and the patients’ symptoms and radiologists’ image impression in free text. These clinical notes were retrieved via Montage (Nuance Communications, Inc, New York, NY). To abstract the presence of a symptom (i.e., pain, fall), we applied regular expressions to the noted indication. To abstract fracture from the physicians’ image interpretation, we used a word2vector-based algorithm previously described by Zech et al.^46^ This supervised learning algorithm required a subset of radiologists’ notes to be manual labeled as either reporting “acute fracture” or “no acute fracture”. Fractures reported anywhere in the image were considered positive (irrespective of anatomic location), and if there was no mention of fracture then the sample was considered an implicit negative. Radiology reports that did not have a corresponding image were used for training the NLP algorithm, which was then used to infer fracture status on the 23,557 matched images and reports used in image model development. We manually reviewed another 100 notes used in image model development to evaluate to performance of the NLP algorithm (Supplementary Table 19). Further label processing was performed to remove infeasible values, binarize values for binary classification models, and impute missing data, as described in the supplementary methods.

### Scalar variable selection

We selected these 20 variables because they have been previously associated with fracture risk (age, gender, and BMI), were common presenting symptoms for patients with hip xrays (fall, pain), have been individually learned in prior image recognition studies (view, radiation dose [OCR]), or mentioned in prior image recognition studies (scanner model, scanner manufacturer, order priority, imaging wait time, time to interpretation, department). We further included HP variables inspired by Agniel et al. (time and date).^18^ The dicom header has hundreds of additional fields, but most are technical minutiae (e.g., table height, distance source to patient) or highly correlated to the aforementioned variables.

### Scalar value filtering

We applied several feasibility filters to remove improbably data values. For any values outside the expected range, we replaced the value with an “NA” indicator. Entries with “NA” values were ignored during the primary regression and logistic modeling, and new values were imputed for secondary analyses as described in “scalar value imputation”. In the clinical note database from Montage, we retrieved the latency times between image order, acquisition, and interpretation. We removed any value that was less than 1 min, or greater than 1 day. Patient BMI values documented greater than 60 were removed.

### Nominal variable consolidation

The hospital has many radiology technicians and physician radiologists who were involved in acquiring and interpreting images in this study, respectively. Because of large imbalances between how many studies each staff member was involved with, we kept the three most common values and consolidated all less frequent values into “other_valid_entry”.

### Scalar variable binarization

For logistic regression models, we coerced continuous or categorical variables into a binary representation. For continuous variables, we simply took the median value and indicated whether values were greater than the median or not. For categorical variables, we used a natural abstraction where possible or took the two most common levels and filling in the remaining entries with “NA” values. For day of week, we abstracted to weekday and weekend. For scan projection, we abstracted to lateral versus bilateral views. For all other categorical variables, we kept the two most common values and removed the others.

### Scalar value imputation

To train multimodal models and perform radiograph case–control matching, we needed to handle missing data in numerous PT and HP variable fields. For categorical variables, missing entries were replaced by an explicit “(Missing)” value. The only PT variable with missing data was BMI. To impute BMI, we trained linear regression models on the subset of data with available BMIs using each combination of predictor variables, possibly with imputed HP variables (see Supplementary Table 20). We used the model with all predictor sets and imputed HP variables to impute all missing BMI entries. For other continuous variables, we simply performed median imputation.

### Model architecture

We used deep-learning models called CNNs to compute abstract image features from input image pixel arrays. Deep-learning models require an abundance of training images to learn meaningful image features during an initial training phase to select parameters that improve a model’s performance on a particular task. We used the inception-v3 CNN architecture^47^ with parameters that have been optimized for natural object recognition in the ImageNet challenge.^48^ We use the pre-trained model to encode radiograph image features and then re-train the final layer, which is a practice called transfer learning and has previously been performed for image recognition tasks in medical radiology.^11–13^ The final classification layer is removed, and we compute the penultimate layer of 2048 image feature scores for each radiograph. We use these abstract feature vectors in subsequent unsupervised models. Deep-learning processing was performed with the python packages keras and tensorflow.

### Unsupervised analysis

After computing the image features for each image, we use several dimensionality reduction techniques to visualize the distribution of image variation. For comparison, we featurized images with one inception-v3 model with randomly initialized parameters and a second that was pre-trained on ImageNet. We performed t-Distributed Stochastic Neighbor Embedding (t-SNE) to project the image feature vector into a 2d plane with the R package Rtsne (initial PCA to 50 dimensions, perplexity 30, theta 0.5, initial momentum 0.5, final momentum 0.8, learning rate 200).

### Supervised analysis

We fine-tune models using image and/or covariate explanatory variable sets and predict binary and continuous outcome variable. For image-only models, we use the 10 principal components of the 2048-dimensional image feature vectors as model input (which describes 68% of image variation in the 2048-d space, see Supplementary Fig. 7), and for combined image–metadata models, we concatenate the 10-principal component image vector with the scalar metadata values. To predict binary variables, we use logistic regression fit to maximize AUC and for continuous variables linear regression to minimize RMSE. This fine-tuning was done in R using the caret package.^49^

### Naive Bayes

Naive Bayes was used to ensemble the predictions of an image-only CNN model with PT or PT + HP variables. The prior probability was estimated using a kernel based on 10-fold cross validation with the training partition. The R package klaR was used to compute the posterior probability of fracture assuming independence between predictors.

### Case–control matching

We create several case–control test cohorts to evaluate the effect of patient matching on fracture detection. Missing data are imputed as discussed in the Supplementary methods. We generate four cohorts by downsampling the images without fracture: random, matched demographics, matched PT, and matched PT + HP (see Supplementary Table 11). For the random case–control group, we randomly select one control for each case. For the three matched cohorts, we compute the dissimilarity between all case–control patient pairs by applying Gower’s method to the pertinent PT and HP covariates and then iteratively select the closest control to each case without replacement.

### Classifier receiver-operating curves

All receiver- operator curves (ROCs) analyses and comparisons were done with the R pROC package.^50^ We compute area under the curve (AUC) with 95% confidence intervals inferred from the DeLong definition for ROC variance.^50^ Comparisons between ROC curves that involved different cohorts (i.e., cross-sectional versus case–control cohort evaluations) were done with unpaired DeLong AUC comparison tests (two-sided, null hypothesis = no difference in AUC). Comparisons between ROC curves that used different predictor sets for the same cohort (i.e., combinatorial IMG, PT, HP predictor models, and Naive Bayes models) used Delong’s paired AUC comparison tests (2-sided, null hypothesis = no difference in AUC). We select the best operating point with Youden’s method to further compute sensitivity, specificity, and other threshold-dependent statistics.

### Odds ratios

Associations between each variable and fracture are performed with the binarized covariates and a two-sided Fisher’s exact test for count data.

## Supplementary information

Supplemental Tables and Figures

## Data Availability

The datasets used in this study are not publicly available because they contain protected health information. Derived and supporting data are available from the corresponding author upon reasonable request.

## Supplementary information

**Supplementary information** accompanies the paper on the *npj Digital Medicine* website (10.1038/s41746-019-0105-1).

## Code availability

R code used in this analysis is available at https://github.com/mbadge/hipsMultimodal.
