# Supplementary material for: Deep learning predicts hip fracture using confounding patient and healthcare variables
Source: NPJ Digit Med. 2019 Apr 30;2:31. doi: 10.1038/s41746-019-0105-1 (PMC6550136; doi:10.1038/s41746-019-0105-1)
Supplement: Supplementary file 1 — Supplemental Tables and Figures [file 41746_2019_105_MOESM1_ESM.pdf]

Supplementary Table 1: Scalar Variable Classifications and Descriptions.

Variable	Class	Description
Fracture	Disease	radiologist documented fracture in the impression report (abstracted with NLP)
Age	Patient	patient age (years)
Gender	Patient	patient's gender
BMI	Patient	patient's body mass index (kg/m2)
Fall	Patient	clinical history of patient falling (abstracted from radiologist's report with regex)
Pain	Patient	clinical history of patient reporting pain (abstracted from radiologist's report with regex)
Department	Hospital Process	hospital setting
Technician	Hospital Process	technician who acquired the radiograph
Radiologist	Hospital Process	radiologist who interpreted the radiograph
Scanner Manufacturer	Hospital Process	company that manufactured the scanner (included in dicom header)
Scanner Model	Hospital Process	device that acquired the radiograph
Time to Initial Interp.	Hospital Process	wait time between image acquisition and the initial interpretation (hours)
Time to Final Interp.	Hospital Process	wait time between image acquisition and the final interpretation (hours)
Order Date	Hospital Process	study day that the image was acquired (days since first scan acquired)
Order Weekday	Hospital Process	day of week of 'Order Date'
Order Time	Hospital Process	time the image was ordered
Order Priority	Hospital Process	whether the order was routine or urgent
Imaging Wait Time	Hospital Process	wait time between image order and image acquisition (hours)
Laterality	Hospital Process	side of patient that was imaged
Radiation Dose	Hospital Process	dose of radiation used (uAs)

Supplementary Table 2: Scalar Variable Representations and Binarization.

**Variable**	**Original Representation (factor levels)**	**Binarization**
Fracture	logical	is fracture?
Age	numeric	age >= 63
Gender	nominal (male, female)	is female?
BMI	numeric	bmi >= 26
Fall	logical	has recent fall?
Pain	logical	has pain?
Department	nominal (emergency department, inpatient, outpatient)	emergency department or inpatient
Technician	nominal (lortiz, sthankachan, technologist, other_valid_entry)	Lortiz or Sthankachan
Radiologist	nominal (alex, darren, sridhar, other_valid_entry)	Darren or Sridhar
Scanner Manufacturer	nominal (Fujifilm, GE, Konica, Philips)	GE or Konica
Scanner Model	nominal (x0862, x5000, bvFamily, bvFamilyXa, cs7, definium5000, discoveryXr656, essentaDr, optimaXr220, thunderPlatform, wdr1)	thunderPlatform or x5000
Time to Initial Interp.	numeric	time >= 114 minutes
Time to Final Interp.	numeric	time >= 202 minutes
Order Date	numeric	>= 4.6 years into the study
Order Weekday	ordinal (Sunday, Monday, Tuesday, ..., Saturday)	weekday (M-F) or weekend (Sa,Su)
Order Time	numeric	time after 1:50pm
Order Priority	logical	is urgent?
Imaging Wait Time	numeric	wait >= 32 minutes
Laterality	nominal (Left, Right, Bilateral)	one-side or bilateral
Radiation Dose	numeric	dose >= 9 uAs

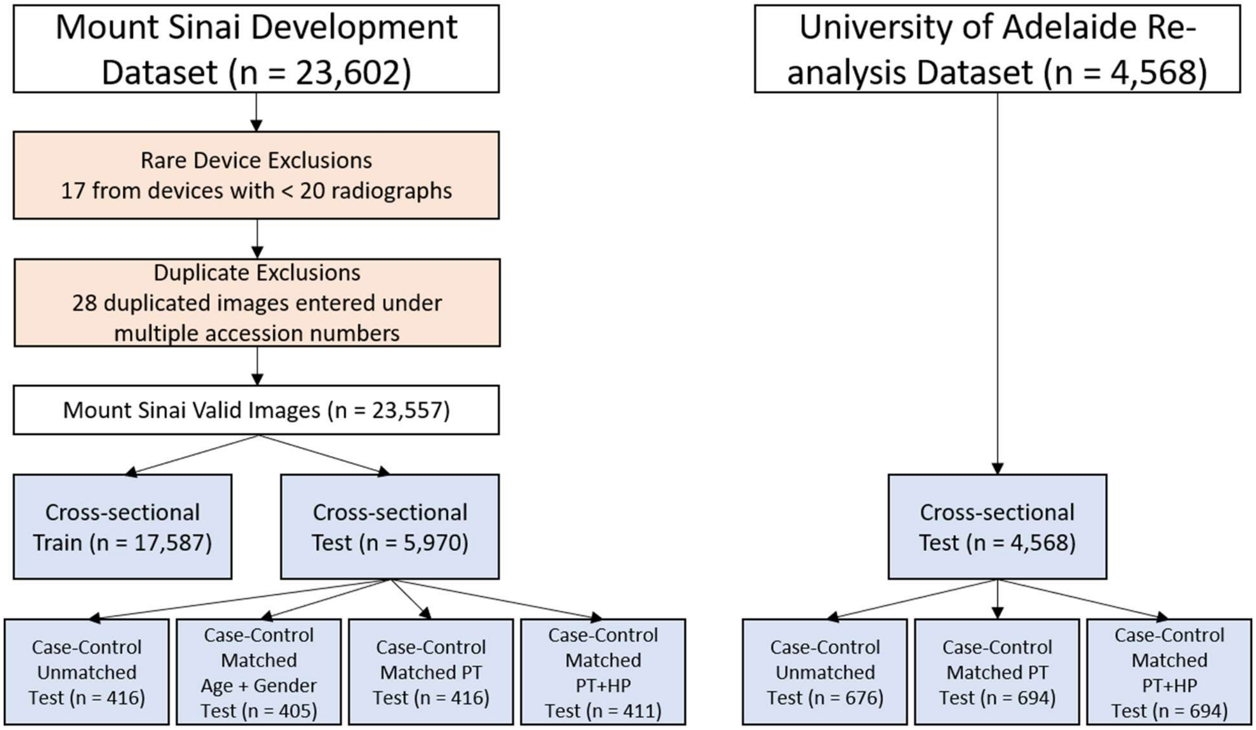

Supplementary Figure 1: Cohort Waterfall Schematic with Preprocessing Exclusions and Subsampling.

Supplementary Table 3: Characteristics of MSH Samples labelled as fracture or normal.

Fracture	FALSE	TRUE
No. radiographs	22,778	779
No. patients	8,736	288
No. scanners	11	7
No. scanner manufacturers	4	4
Age, mean (SD), years	61 (22)	74 (22)
Female frequency, No. (%)	15,022 (66)	498 (64)
BMI, mean (SD)	28 (7)	24 (5)
Fall frequency, No. (%)	4,062 (18)	291 (37)
Pain frequency, No. (%)	11,745 (52)	225 (29)

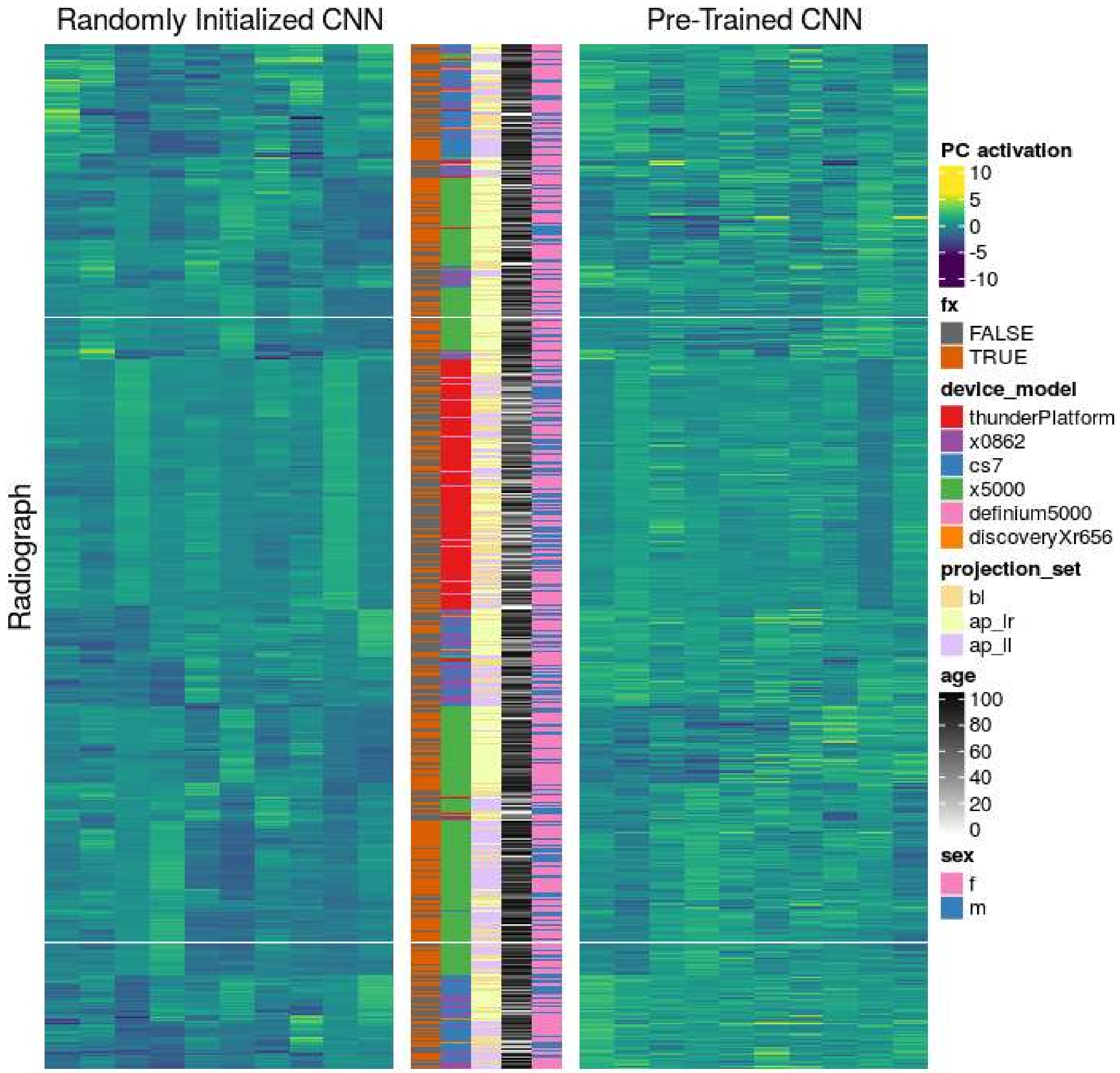

Supplementary Figure 2: Image Feature Matrix annotated with fracture and covariates. The data is represented by a row for each radiograph and a column for each CNN principle component feature or scalar feature. On the left are image principal components computed with a randomly initialized CNN, and on the right are image principal components computed with a CNN pre-trained on ImageNet. The PC activation fill reflects the neural activation of each feature for each radiograph. Radiographs are clustered and annotated with fracture and several covariates. For this figure samples were enriched for fracture by randomly sampling 500 images with and 500 without fracture.

Supplementary Table 4: Strongest covariate predictor for each image principal component. Univariate linear regression models were trained to predict each image component with each explanatory covariate, and shown here are the highest scoring predictors for each image component.

**Explanatory Covariate**	**Image Component**	R^2
Scanner Model	PC1	0.59
Scanner Model	PC2	0.52
Scanner Model	PC3	0.65
Scanner Model	PC4	0.03
Scanner Model	PC5	0.07
Technician	PC6	0.04
Scanner Model	PC7	0.06

Supplementary Table 5: Performance of image models predicting each binarized variable. CNN image features were used to train logistic regression models on binarized forms of each scalar variable and various performance metrics were computed. The threshold column displays the decision cutoff used to compute all the operating point dependent statistics. The AUC 95% confidence interval was determined by DeLong definition for AUC variance.

**Classification target**	**auc**	**auprc**	**threshold**	**spec**	**sens**	**acc**	**npv**	**ppv**	**tn**	**tp**	**fn**	**fp**
Scanner Model	1.00 (1.00-1.00)	1.00	0.283	0.99	1.00	1.00	1.00	0.992	1,767	1,265	3	10
Scanner Manufacturer	0.98 (0.98-0.99)	0.98	0.299	0.92	0.98	0.95	0.98	0.928	2,048	2,283	41	178
Order Priority	0.79 (0.77-0.80)	0.57	0.255	0.56	0.92	0.68	0.93	0.523	2,203	1,880	169	1,718
Fracture	0.78 (0.74-0.81)	0.11	0.033	0.74	0.74	0.74	0.99	0.094	4,283	153	54	1,480
Order Date	0.77 (0.76-0.79)	0.72	0.522	0.65	0.78	0.72	0.73	0.707	1,867	2,417	682	1,004
Technician	0.72 (0.66-0.78)	0.28	0.138	0.57	0.77	0.60	0.94	0.223	219	48	14	167
Order Weekday	0.71 (0.69-0.72)	0.95	0.930	0.97	0.41	0.47	0.18	0.991	664	2,161	3,125	20
Radiologist	0.70 (0.67-0.72)	0.59	0.446	0.56	0.74	0.63	0.77	0.528	611	538	186	480
Radiation Dose	0.70 (0.67-0.72)	0.64	0.490	0.69	0.63	0.66	0.71	0.608	900	616	368	397
Age	0.67 (0.65-0.68)	0.65	0.485	0.62	0.64	0.63	0.64	0.630	1,859	1,920	1,061	1,130
Fall	0.67 (0.65-0.68)	0.28	0.140	0.45	0.85	0.52	0.93	0.266	2,163	968	171	2,668
Time to Final Interp.	0.65 (0.63-0.66)	0.60	0.500	0.55	0.71	0.63	0.66	0.603	1,614	2,004	830	1,322
Department	0.63 (0.61-0.65)	0.50	0.334	0.63	0.58	0.61	0.72	0.475	1,241	652	472	720
Laterality	0.63 (0.61-0.64)	0.51	0.382	0.56	0.64	0.59	0.70	0.487	2,027	1,505	850	1,588
Time to Initial Interp.	0.63 (0.60-0.66)	0.63	0.573	0.79	0.44	0.62	0.59	0.668	504	274	347	136
Pain	0.62 (0.61-0.64)	0.59	0.475	0.47	0.72	0.59	0.63	0.572	1,404	2,144	816	1,606
BMI	0.62 (0.60-0.64)	0.58	0.522	0.56	0.64	0.60	0.62	0.588	780	872	484	612
Imaging Wait Time	0.61 (0.60-0.63)	0.60	0.435	0.46	0.73	0.60	0.62	0.586	1,112	1,870	680	1,322
Order Time	0.58 (0.57-0.60)	0.59	0.496	0.52	0.60	0.56	0.56	0.569	1,523	1,840	1,212	1,395
Gender	0.55 (0.54-0.57)	0.40	0.347	0.64	0.45	0.57	0.68	0.401	2,473	937	1,160	1,400

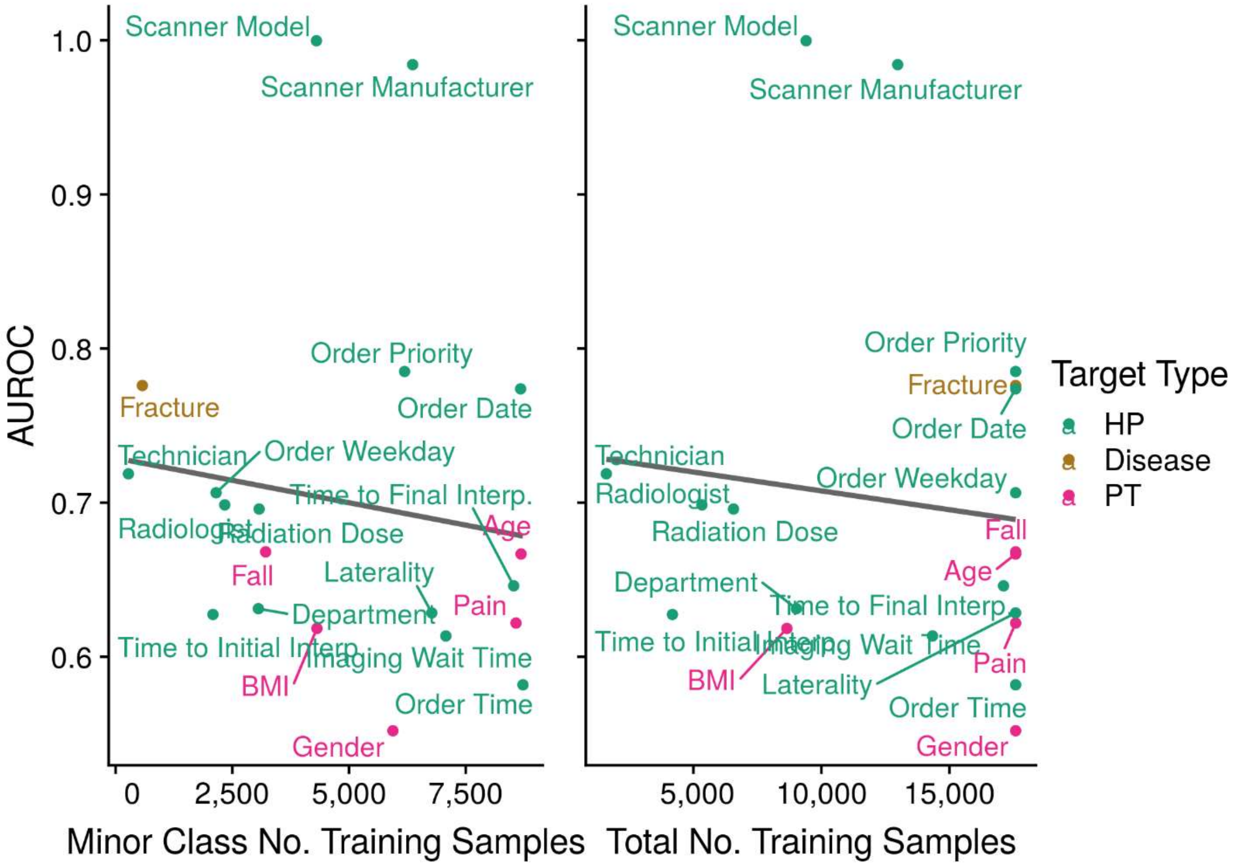

Supplementary Figure 3: Sample size is not the primary determinant of model performance. This is a new view into the data displayed in Figure 2A. Here we plot classification performance for each target against two metrics for the amount of training data. On the right, we show performance versus the total number of training samples (e.g., the number of non-missing values), and on the left, we show performance versus the number of examples for the class with less samples in order to account for the substantial class imbalance for labels like fracture. The grey line is a linear regression of performance versus sample size across all model targets.

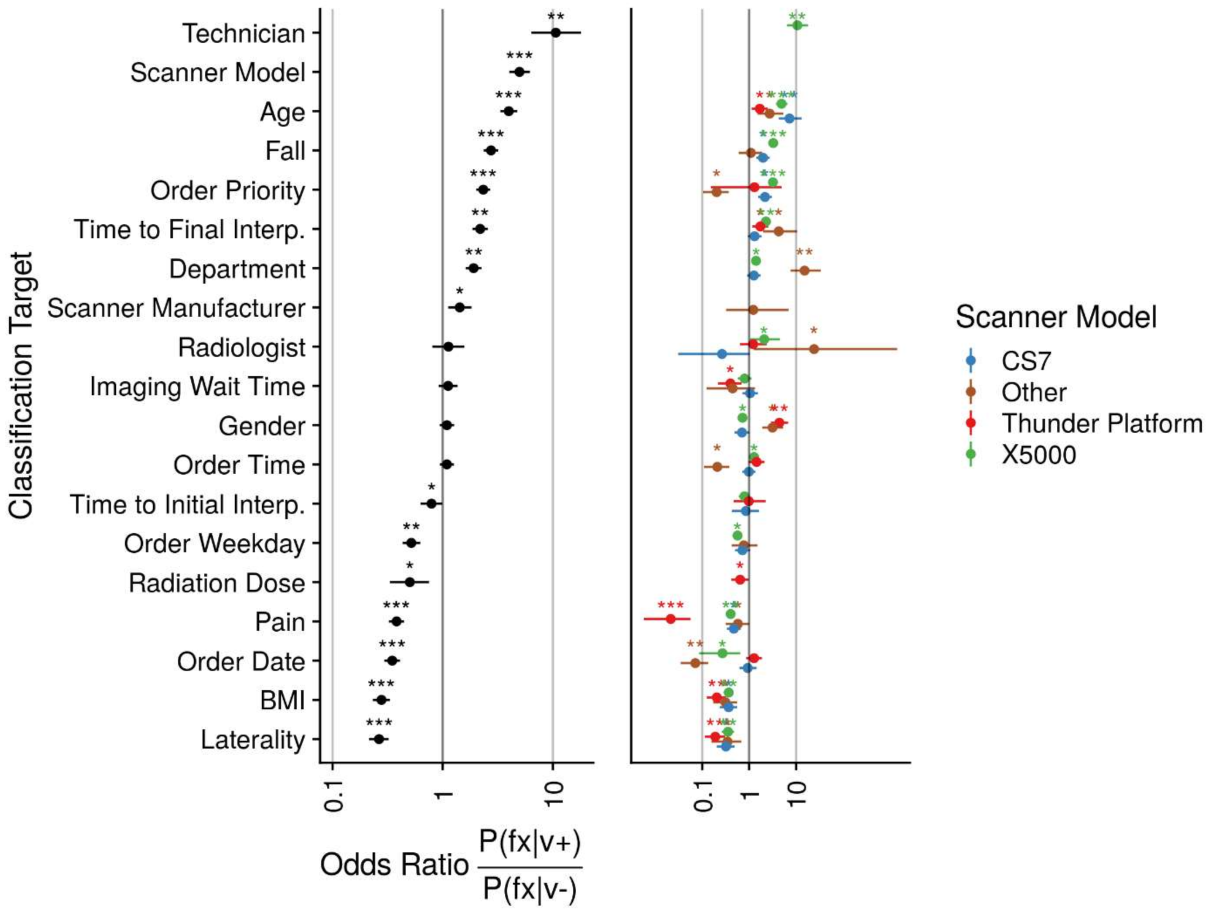

Supplementary Figure 4: Association between fracture and covariates. Univariate associations between hip fracture and each covariate were assessed using Fisher's Exact test on the full dataset (left) and after stratifying by the scanner device (right). Each covariate was binarized as described in the supplemental methods. Significance indicators: * = $p<0.05$, ** = $p<1e-10$, and *** = $p<1e-25$.

Supplementary Table 6: Performance of Image Models Predicting each Continuous Variable. Image features were used to train regression models on each of the 6 continuous covariates, and R^2 values were computed on the test-set.

**Classification Target**	**R^2**
Order Date	0.39
Radiation Dose	0.13
Age	0.07
Time to Final Interpretation	0.06
BMI	0.06
Imaging Wait Time	0.03
Order Time	0.02
Time to Initial Interpretation	0.02

Supplementary Table 7: Predicting fracture with combinations of radiographs, patient and hospital process covariates. Various performance metrics for logistic regression models. The AUC 95% confidence interval was determined by DeLong definition for AUC variance.

Predictor Set	auc	auprc	threshold	spec	sens	acc	npv	ppv	tn	tp	fn	fp
IMG + PT + HP	0.91 (0.90-0.93)	0.40	0.048	0.85	0.83	0.85	0.99	0.168	4,910	172	35	853
HP	0.89 (0.87-0.91)	0.37	0.037	0.78	0.86	0.78	0.99	0.121	4,478	177	30	1,285
IMG + HP	0.89 (0.87-0.91)	0.38	0.054	0.84	0.77	0.84	0.99	0.148	4,844	160	47	919
PT + HP	0.87 (0.85-0.89)	0.14	0.033	0.75	0.89	0.76	0.99	0.115	4,339	185	22	1,424
IMG + PT	0.86 (0.83-0.88)	0.24	0.045	0.83	0.78	0.83	0.99	0.143	4,797	161	46	966
PT	0.79 (0.75-0.82)	0.15	0.028	0.62	0.83	0.63	0.99	0.072	3,567	171	36	2,196
IMG	0.78 (0.74-0.81)	0.11	0.033	0.74	0.74	0.74	0.99	0.094	4,283	153	54	1,480

Supplementary Table 8: Comparison of fracture detection models trained on image and/or patient and hospital process factors. All test sets are shared, so DeLong paired test was performed to assess for an AUROC difference for each pair of predictors.

Classifier 1	Classifier 2	p-value, DeLong paired AUC comparison
HP	IMG + HP	0.967
IMG	PT	0.652
PT + HP	IMG + PT	0.045
PT + HP	IMG + HP	0.006
IMG + PT	IMG + HP	0.002
HP	PT + HP	0.002
HP	IMG + PT	0.001
IMG + HP	IMG + PT + HP	1e-06
HP	IMG + PT + HP	3e-07
IMG	IMG + PT	1e-09
IMG	PT + HP	1e-10
PT	IMG + HP	2e-11
PT	HP	9e-12
PT	PT + HP	8e-13
PT	IMG + PT	2e-13
IMG + PT	IMG + PT + HP	2e-14
IMG	HP	3e-17
IMG	IMG + HP	5e-18
IMG	IMG + PT + HP	1e-21
PT	IMG + PT + HP	3e-27
PT + HP	IMG + PT + HP	4e-45

Supplementary Table 9: Performance of an image-based fracture detection model evaluated on test-sets with variable case-control sampling strategies. The AUC 95% confidence interval was determined by DeLong definition for AUC variance.

Test Cohort	auc	auprc	threshold	spec	sens	acc	npv	ppv	tn	tp	fn	fp
Cross Sectional	0.78 (0.74-0.81)	0.11	0.033	0.74	0.74	0.74	0.99	0.094	4,283	153	54	1,480
Case Control, no matching	0.77 (0.73-0.82)	0.74	0.032	0.77	0.75	0.76	0.75	0.760	160	155	52	49
Case Control, matched Age, Gender	0.76 (0.71-0.81)	0.73	0.029	0.66	0.78	0.72	0.74	0.703	130	161	46	68
Case Control, matched PT	0.67 (0.62-0.72)	0.65	0.032	0.55	0.75	0.65	0.69	0.620	114	155	52	95
Case Control, matched PT + HP	0.53 (0.47-0.59)	0.54	0.033	0.37	0.74	0.56	0.58	0.544	76	153	54	128

Supplementary Table 10: Comparison of a fracture detection model evaluated on differentially sampled test cohorts.

Classifier 1	Classifier 2	p-value, DeLong unpaired AUC comparison
Cross Sectional	Case Control, no matching	0.961
Case Control, no matching	Case Control, matched Age, Gender	0.652
Cross Sectional	Case Control, matched Age, Gender	0.571
Case Control, matched Age, Gender	Case Control, matched PT	0.013
Case Control, no matching	Case Control, matched PT	0.003
Cross Sectional	Case Control, matched PT	0.000
Case Control, matched PT	Case Control, matched PT + HP	0.000
Case Control, matched Age, Gender	Case Control, matched PT + HP	1e-09
Case Control, no matching	Case Control, matched PT + HP	5e-11
Cross Sectional	Case Control, matched PT + HP	1e-13

Supplementary Table 11: Variables used for case-control matching in each dataset.

Dataset	Matching	Matched Variables
Adelaide	Random	N/A
Adelaide	PT	Age and Gender
Adelaide	PT+HP	Age, Gender, Scanner Model, Scanner Manufacturer, Radiologist and Order Weekday
Mount Sinai	Random	N/A
Mount Sinai	dem	Age and Gender
Mount Sinai	PT	Age, Gender, BMI, Fall and Pain
Mount Sinai	PT+HP	Age, Gender, BMI, Fall, Pain, Scanner Model, Scanner Manufacturer, Radiologist, Order Weekday, Department, Laterality, Order Date, Order Time, Technician, Radiation Dose, Imaging Wait Time, Time to Initial Interp. and Time to Final Interp.

Supplementary Table 12: Population Characteristics of Adelaide Test Cohorts after Subsampling with Variable Matching.

**Cohort**	**crossSectional**	**caseControl matchAll**	**caseControl matchDem**	**caseControl matchNone**
Sampling	Cross-Sectional	Case-Control	Case-Control	Case-Control
Matching	NA	PT + HP	PT	NA
Partition	Test	Test	Test	Test
No. radiographs	4,568	694	694	676
No. scanners	14	13	13	14
No. scanner manufacturers	7	6	6	7
Age, mean (SD), years	57 (25)	81 (14)	81 (14)	68 (24)
Fracture frequency, No. (%)	347 (8)	347 (50)	347 (50)	347 (51)
Female frequency, No. (%)	2,135 (47)	458 (66)	456 (66)	388 (57)

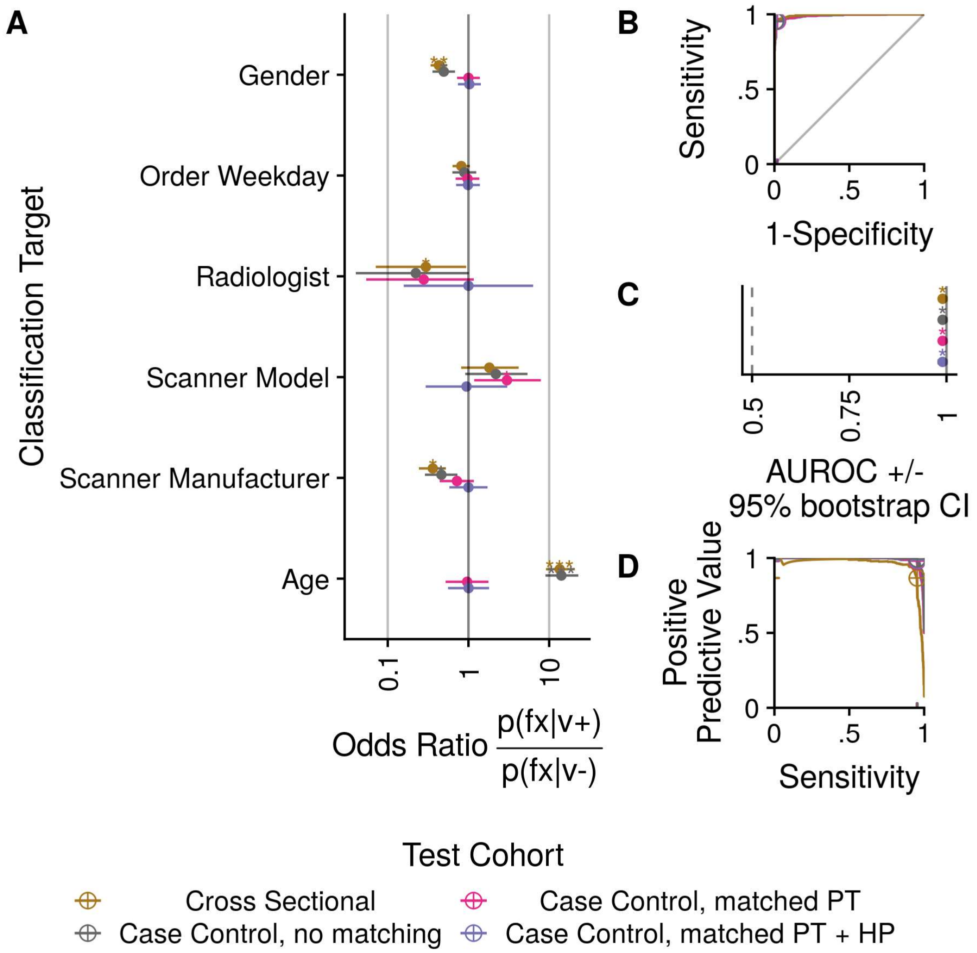

Supplementary Figure 5: Association of covariates and fracture and the performance of fracture detection models evaluated on differentially sampled test cohorts from the Adelaide dataset. A) The association between each covariate and fracture, colored by how the test cohort is sampled. (*) indicate a Fisher's Exact test with $p<0.05$. B) ROC and D) Precision Recall curves for the image-classifier tested on differentially sampled test sets. The best operating point is indicated with crosshairs. (*) represents a 95% confidence interval that does not include 0.5. C) Summary of (B) with 95% bootstrap confidence intervals.

Supplementary Table 13: Performance of fracture detection models evaluated on differentially sampled test cohorts from the Adelaide dataset. The AUC 95% confidence interval was determined by DeLong definition for AUC variance.

Test Cohort	auc	auprc	threshold	spec	sens	acc	npv	ppv	tn	tp	fn	fp
Cross Sectional	0.99 (0.99-1)	0.96	0.60	0.99	0.95	0.99	1.00	0.87	4,170	331	16	51
Case Control, matched PT	0.99 (0.99-1)	0.99	0.59	0.98	0.95	0.97	0.96	0.98	340	331	16	7
Case Control, no matching	0.99 (0.99-1)	1.00	0.60	0.99	0.95	0.97	0.95	0.99	327	331	16	2
Case Control, matched PT + HP	0.99 (0.98-1)	0.99	0.57	0.99	0.95	0.97	0.96	0.99	342	331	16	5

Supplementary Table 14: Comparison of fracture detection models evaluated on differentially sampled test cohorts from the Adelaide dataset.

Classifier 1	Classifier 2	p-value, DeLong unpaired AUC comparison
Cross Sectional	Case Control, no matching	0.75
Case Control, matched PT	Case Control, matched PT + HP	0.73
Cross Sectional	Case Control, matched PT	0.33
Case Control, matched PT	Case Control, no matching	0.23
Cross Sectional	Case Control, matched PT + HP	0.20
Case Control, matched PT + HP	Case Control, no matching	0.14

Supplementary Table 15: Comparing the performance of models trained directly on different predictor sets, and models that ensemble image models with covariates. Each primary model is a logistic regression model to predict fracture. Naive Bayes ensembles were constructed to combine evidence from the image model and other predictor sets without knowing the interdependencies between them. The AUC 95% confidence interval was determined by DeLong definition for AUC variance.

Classifier	auc	auprc	threshold	spec	sens	acc	npv	ppv	tn	tp	fn	fp
pt	0.79 (0.75-0.82)	0.15	0.028	0.62	0.83	0.63	0.99	0.072	3,567	171	36	2,196
ptHp	0.87 (0.85-0.89)	0.14	0.033	0.75	0.89	0.76	0.99	0.115	4,339	185	22	1,424
imgPt	0.86 (0.83-0.88)	0.24	0.045	0.83	0.78	0.83	0.99	0.143	4,797	161	46	966
imgPtHp	0.91 (0.90-0.93)	0.40	0.048	0.85	0.83	0.85	0.99	0.168	4,910	172	35	853
nb_imgPtHp	0.90 (0.88-0.93)	0.33	0.052	0.86	0.82	0.86	0.99	0.175	4,969	169	38	794
nb_imgPt	0.84 (0.81-0.87)	0.22	0.034	0.83	0.77	0.82	0.99	0.138	4,764	160	47	999

Supplementary Table 16: Statistical comparison of pairs of multimodal and naive Bayes models to predict fracture.

Classifier 1	Classifier 2	p-value, DeLong paired AUC comparison
pt	img_pt	2e-13
pt	nb_img_pt	2e-08
pt_hp	img_pt_hp	4e-45
pt_hp	nb_img_pt_hp	5e-11
img_pt	nb_img_pt	0.014
img_pt_hp	nb_img_pt_hp	0.003

Supplementary Table 17: Characteristics of MSH Samples stratified by the scanner that captured the image

Scanner	No. radiographs	No. patients	Age, mean (SD), years	Female frequency, No. (%)	Fracture frequency, No. (%)	BMI, mean (SD)	Fall frequency, No. (%)	Pain frequency, No. (%)
thunderPlatform	6,879	1,940	54 (18)	4,506 (66)	114 (1.7)	29 (7)	280 (4)	4,266 (62)
x5000	5,570	2,507	62 (25)	3,763 (68)	431 (7.7)	26 (7)	1,334 (24)	3,020 (54)
x0862	5,106	2,053	68 (20)	3,339 (65)	11 (0.2)	27 (6)	1,497 (29)	1,397 (27)
cs7	3,836	1,908	63 (25)	2,520 (66)	162 (4.2)	26 (6)	1,035 (27)	2,024 (53)
definium5000	957	384	60 (16)	617 (64)	0 (0.0)	28 (7)	80 (8)	657 (69)
discoveryXr656	516	225	64 (24)	308 (60)	3 (0.6)	27 (6)	85 (16)	247 (48)
essentaDr	357	245	59 (18)	279 (78)	4 (1.1)	28 (6)	9 (3)	218 (61)
wdr1	125	45	60 (16)	81 (65)	0 (0.0)	27 (4)	2 (2)	96 (77)
optimaXr220	112	52	72 (18)	68 (61)	0 (0.0)	27 (5)	19 (17)	33 (29)
bvFamily	79	12	78 (14)	29 (37)	54 (68.4)	23 (4)	12 (15)	12 (15)
bvFamilyXa	20	6	43 (39)	10 (50)	0 (0.0)	18 (3)	0 (0)	0 (0)

Supplementary Table 18: Characteristics of MSH Samples stratified by department

Department	Emergency Department	Inpatient	Outpatient	(Missing)
No. radiographs	7,926	4,182	3,444	8,005
No. patients	3,676	1,720	1,686	2,421
Age, mean (SD), years	65 (24)	69 (20)	58 (22)	54 (19)
Female frequency, No. (%)	5,377 (68)	2,559 (61)	2,319 (67)	5,265 (66)
Fracture frequency, No. (%)	318 (4.0)	309 (7.4)	28 (0.8)	124 (1.5)
BMI, mean (SD)	26 (7)	26 (6)	28 (6)	29 (7)
Fall frequency, No. (%)	2,661 (34)	1,118 (27)	232 (7)	342 (4)
Pain frequency, No. (%)	3,527 (44)	1,501 (36)	2,135 (62)	4,807 (60)

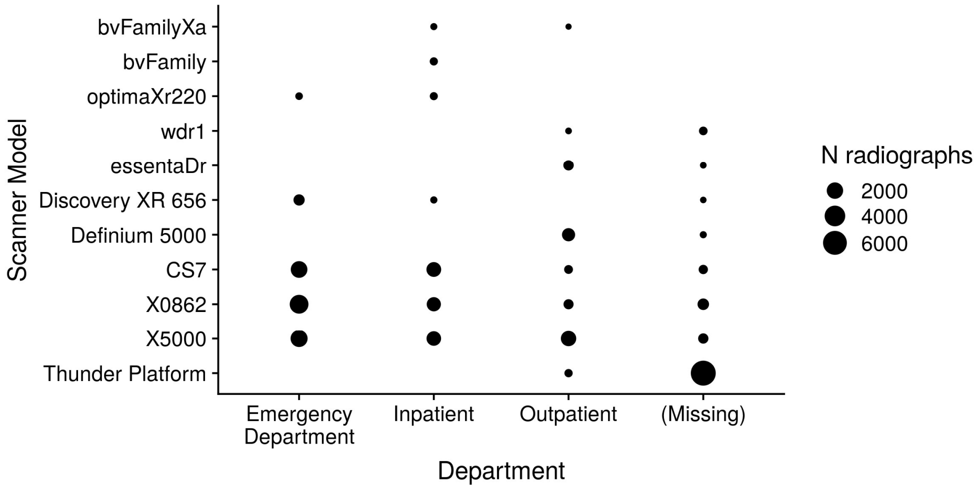

Supplementary Figure 6: Bivariate distribution of radiographs collected from different scanners and departments.

Supplementary Table 19: Performance of Natural Language Processing (NLP) abstraction of radiologists' image impressions. PPV = positive predictive value, NPV = negative predictive value, FPR = false positive rate.

Accuracy	Sensitivity	Specificity	PPV	NPV	FPR
0.8	0.89	0.74	0.68	0.92	0.26

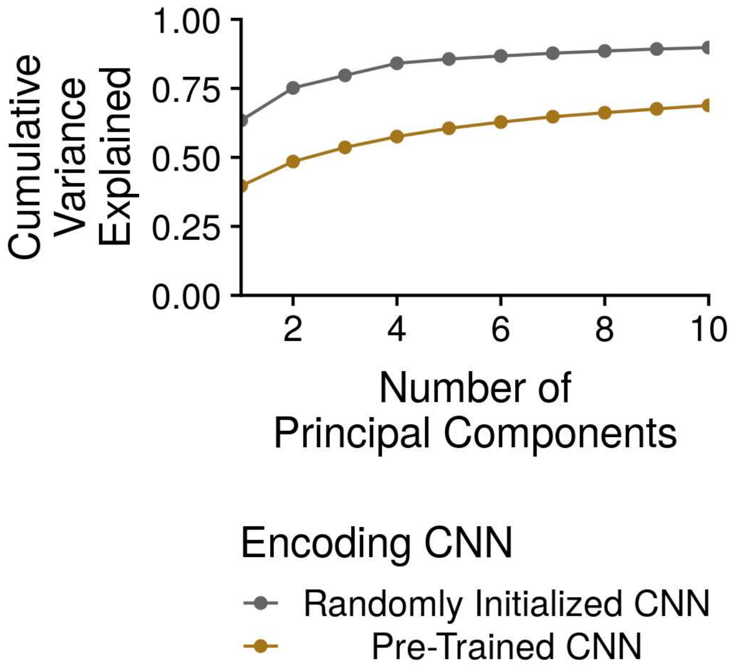

Supplementary Figure 7: Cumulative Variance Explained after CNN Feature Dimensionality Reduction. 69% of image variance in 2,048 CNN features is captured by 10 principal components.

Supplementary Table 20: Performance of BMI Imputation with different predictor sets.

Predictor Set	Imputed HP variables	RMSE	R^2	RMSE SD	R^2 SD
IMG	FALSE	6.6	0.06	0.06	0.005
PT	FALSE	6.7	0.03	0.07	0.004
HP	FALSE	6.6	0.05	0.08	0.005
IMG + PT	FALSE	6.5	0.08	0.06	0.006
IMG + HP	FALSE	6.5	0.08	0.06	0.007
IMG + PT + HP	FALSE	6.5	0.09	0.06	0.008
HP	TRUE	6.3	0.14	0.07	0.007
IMG + HP	TRUE	6.2	0.17	0.06	0.009
IMG + PT + HP	TRUE	6.1	0.18	0.06	0.010
